# Supplementary material for: De novo biosynthesis of antiarrhythmic alkaloid ajmaline
Source: Nat Commun. 2024 Jan 11;15:457. doi: 10.1038/s41467-024-44797-z (PMC10784492; doi:10.1038/s41467-024-44797-z)
Supplement: Supplementary file 1 — Supplementary Information [file 41467_2024_44797_MOESM1_ESM.pdf]

De novo biosynthesis of antiarrhythmic alkaloid ajmaline

Guo *et al.*

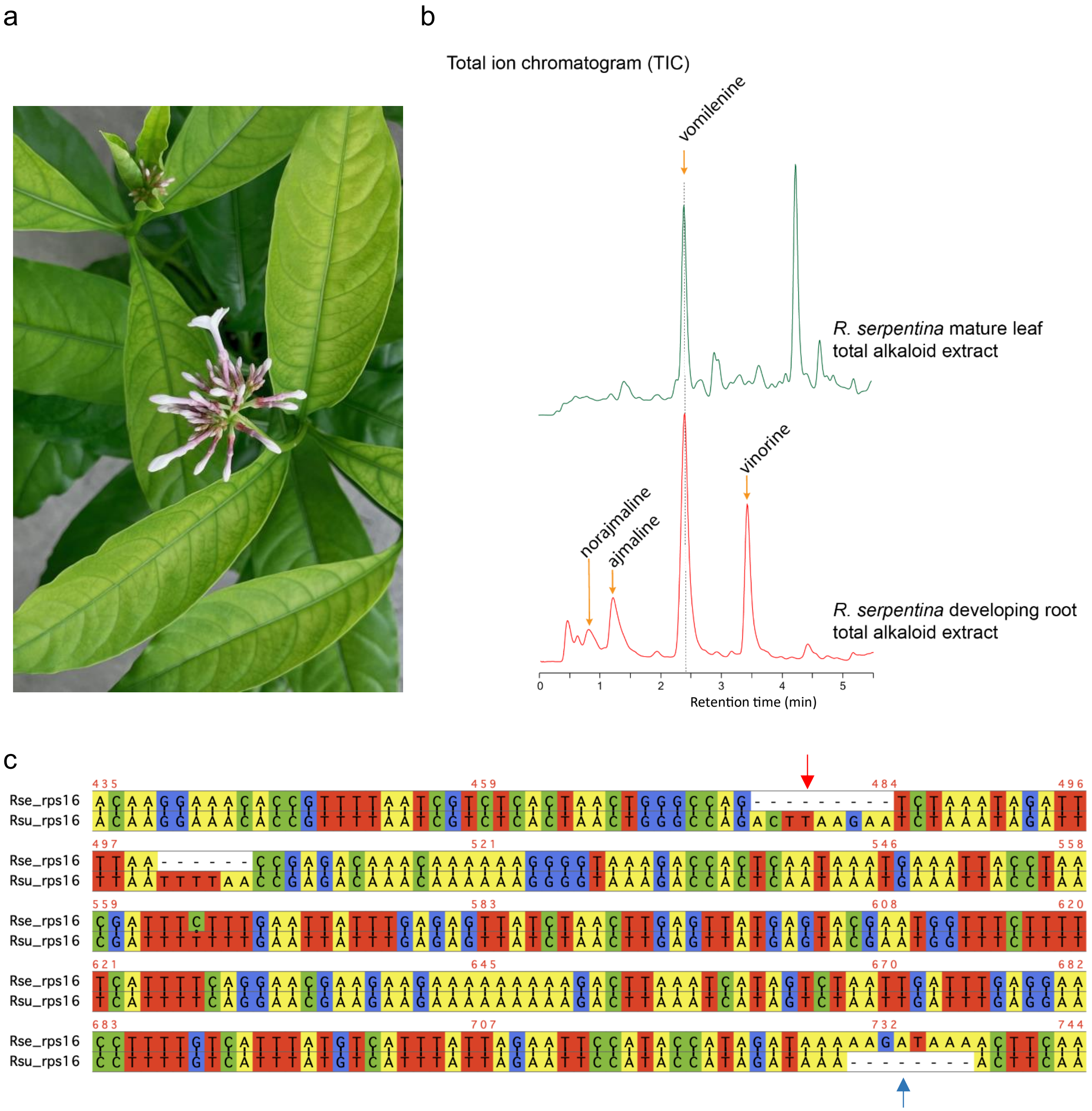

Supplementary Figure 1. Vomilenine was detected in both leaf and developing root tissues of the plant species *Rauvolfia serpentina*. (**a**) Greenhouse grown *R. serpentina* plant used in this study. (**b**) The LC-MS/MS total ion chromatogram (TIC) showed that vomilenine accumulated as one of the major alkaloids in these tissues. Ajmaline and norajmaline were not detected in leaf but in root. (**c**) Alignment of partial chloroplast *rps16* (16S ribosomal RNA encoding gene) cloned from *R. serpentina* plant used in this study and a reference *rps16* gene of *R. sumatrana*. The red arrow indicates the 9 bp deletion and the blue arrow indicates the 8 bp insertion. Both the deletion and insertion are unique to *R. serpentina* when compared to the *rps16* gene from 38 other Rauvolfia species [1].

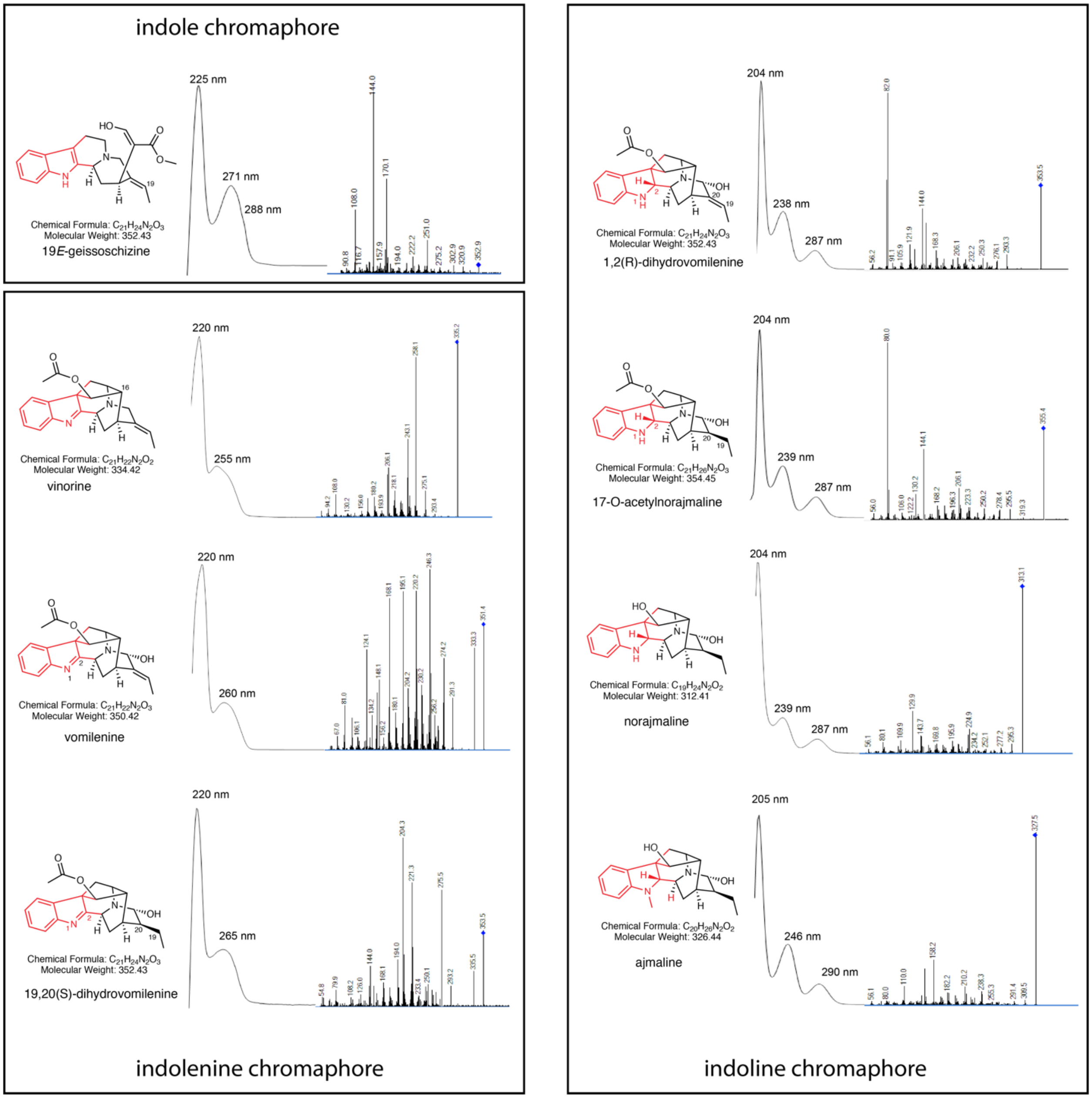

Supplementary Figure 2. The UV absorption profiles and ESI-MS/MS ion fragmentation patterns of the alkaloids in this study. The structures in red are the chromophores responsible for their unique UV absorption profiles, which are distinct for indole (geissoschizine), indoline (1,2-dihydrovomilenine, 17-*O*-acetylnorajmaline, norajmaline and ajmaline), and indolenine (vinorine, vomilenine, 19,20-dihydrovomilenine) chromophores. Norajmaline *N*-methylation results in absorption shift to longer wavelengths.

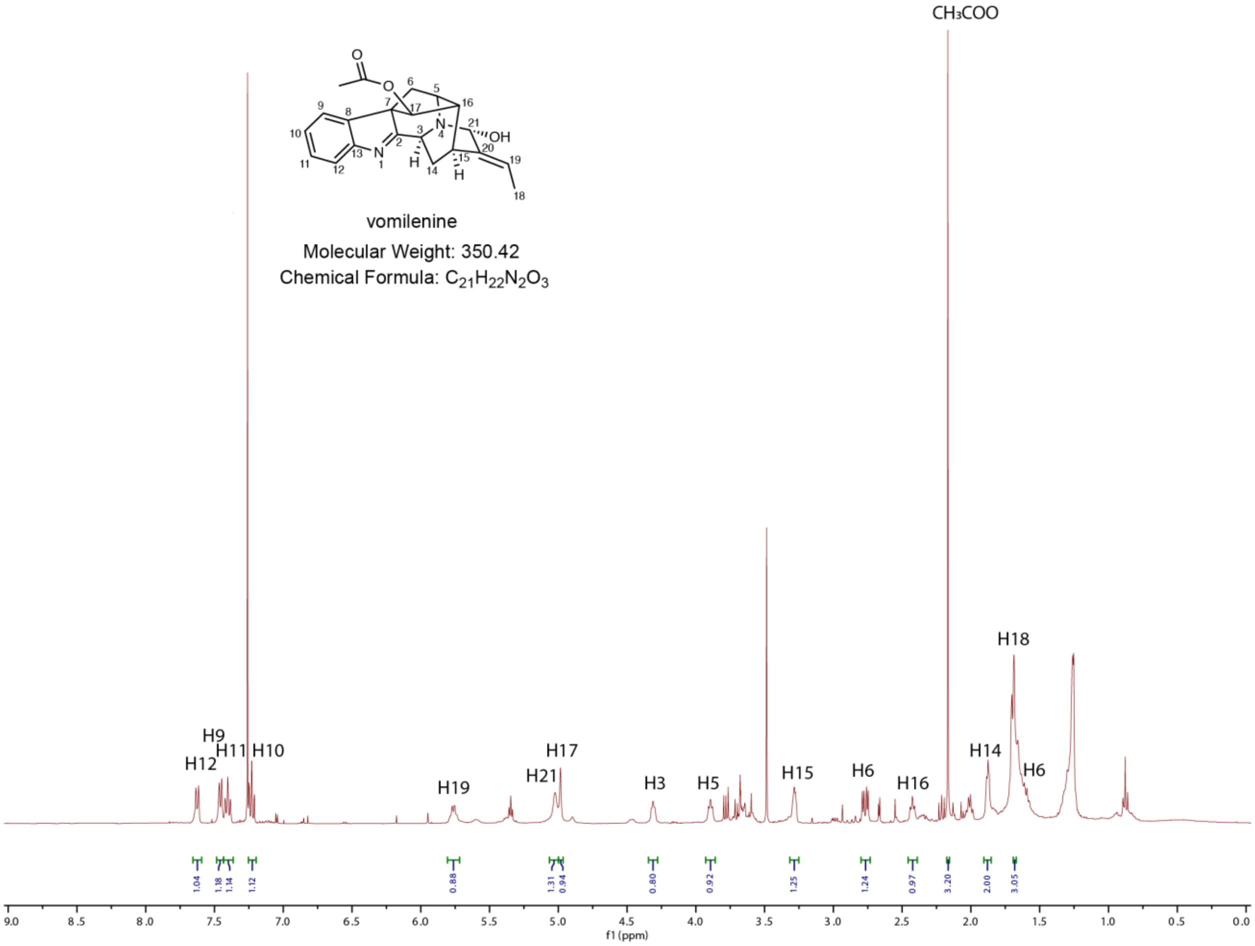

Supplementary Figure 3. **^{1}H-NMR spectra of vomilenine in $CDCl_3$.**

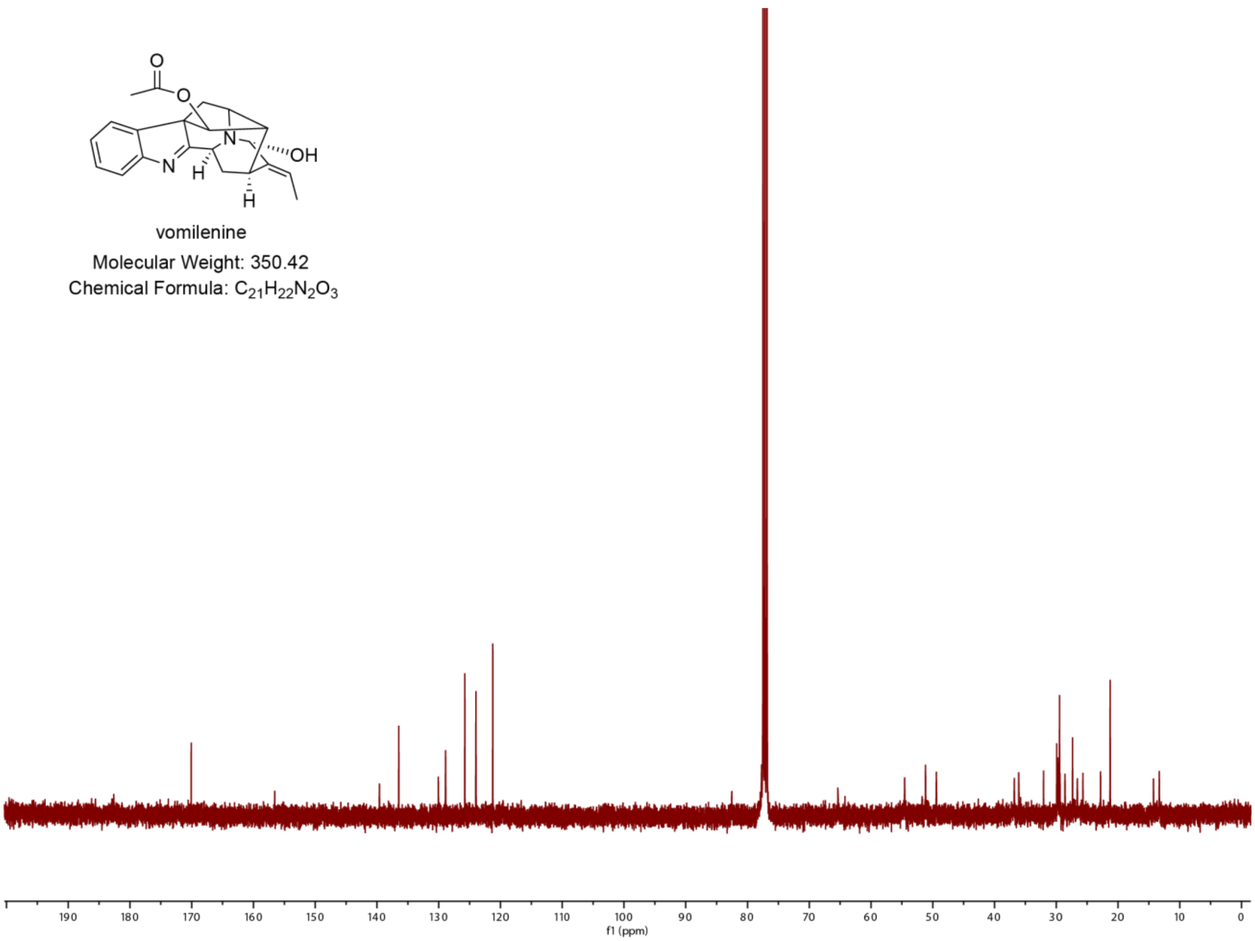

Supplementary Figure 4. **^{13}C-NMR spectra of vomilenine in $CDCl_3$.**

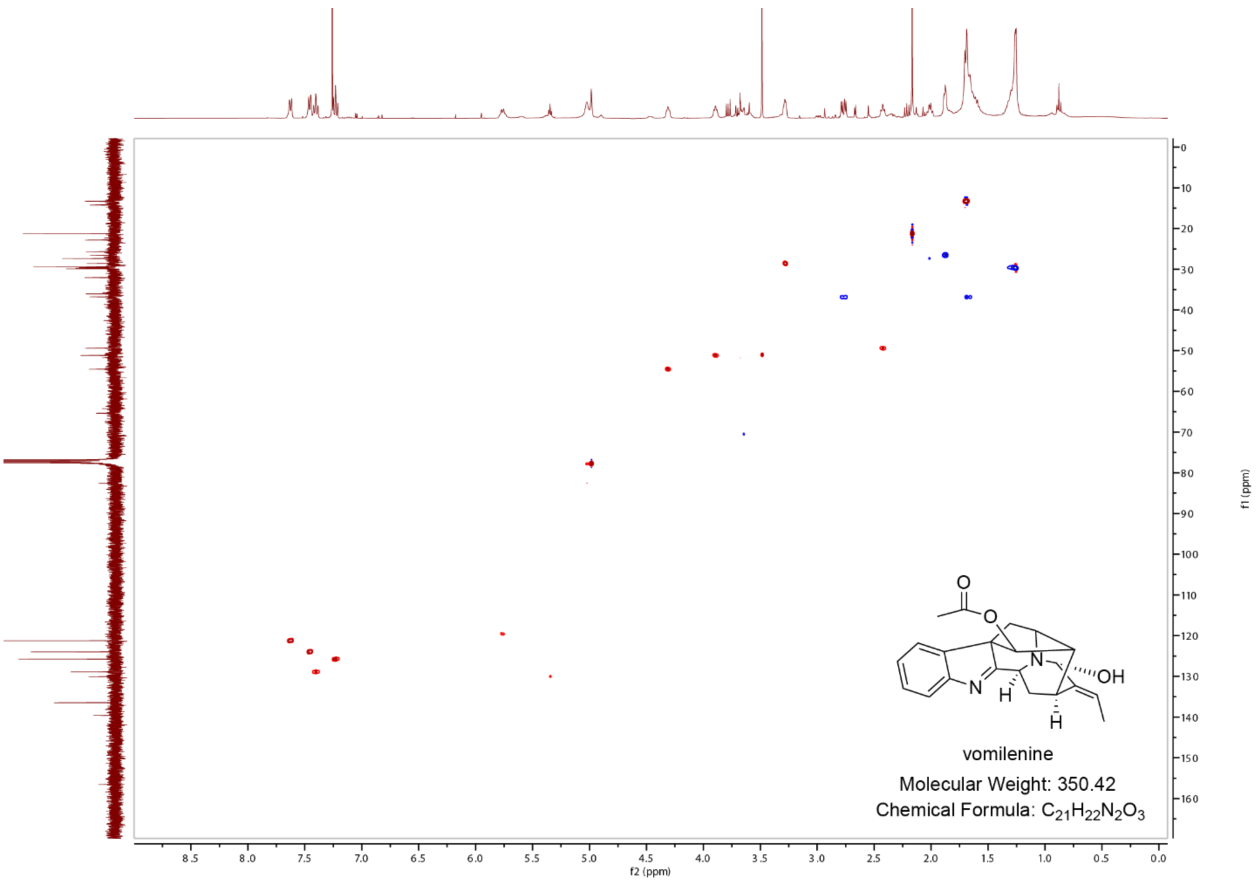

Supplementary Figure 5. HSQC spectra of vomilenine in $CDCl_3$.

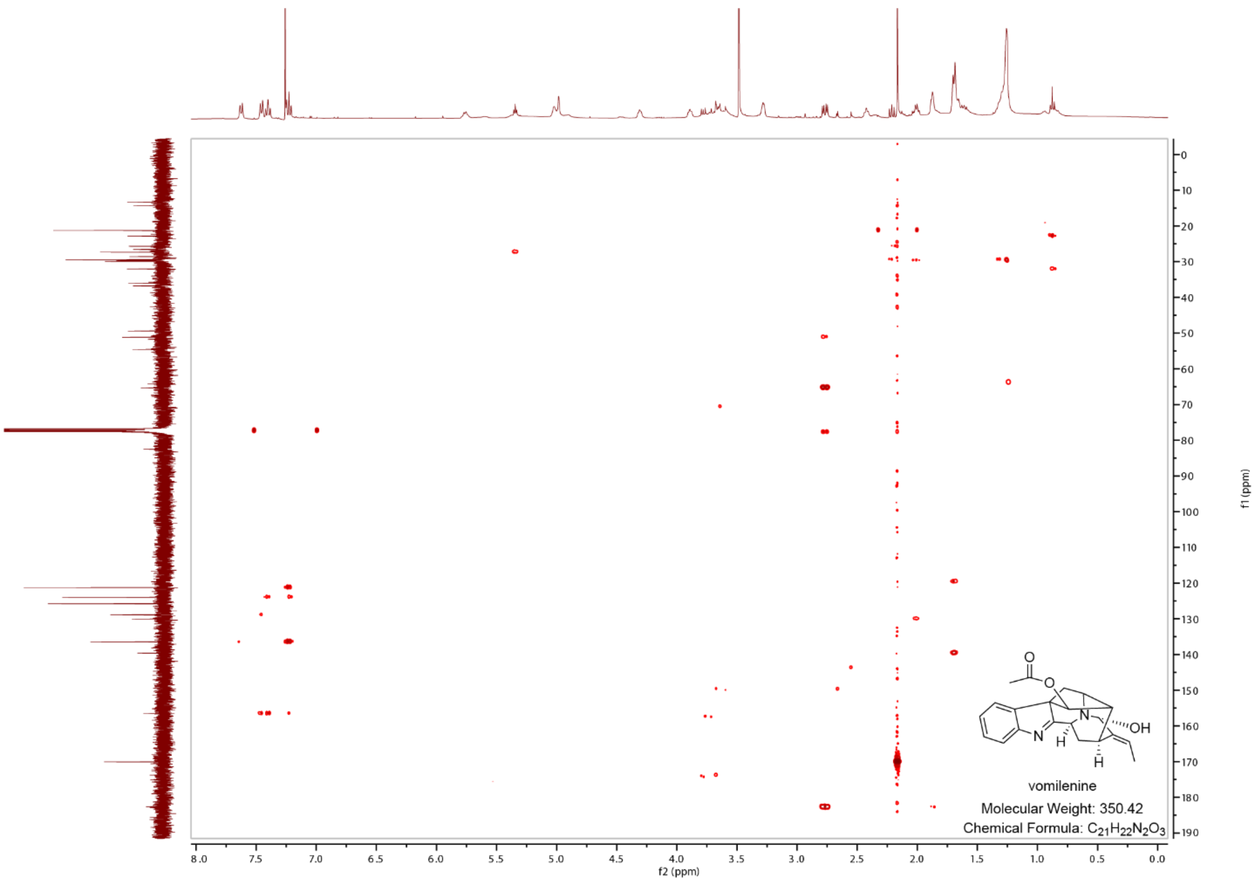

Supplementary Figure 6. HMBC spectra of vomilenine in $CDCl_3$.

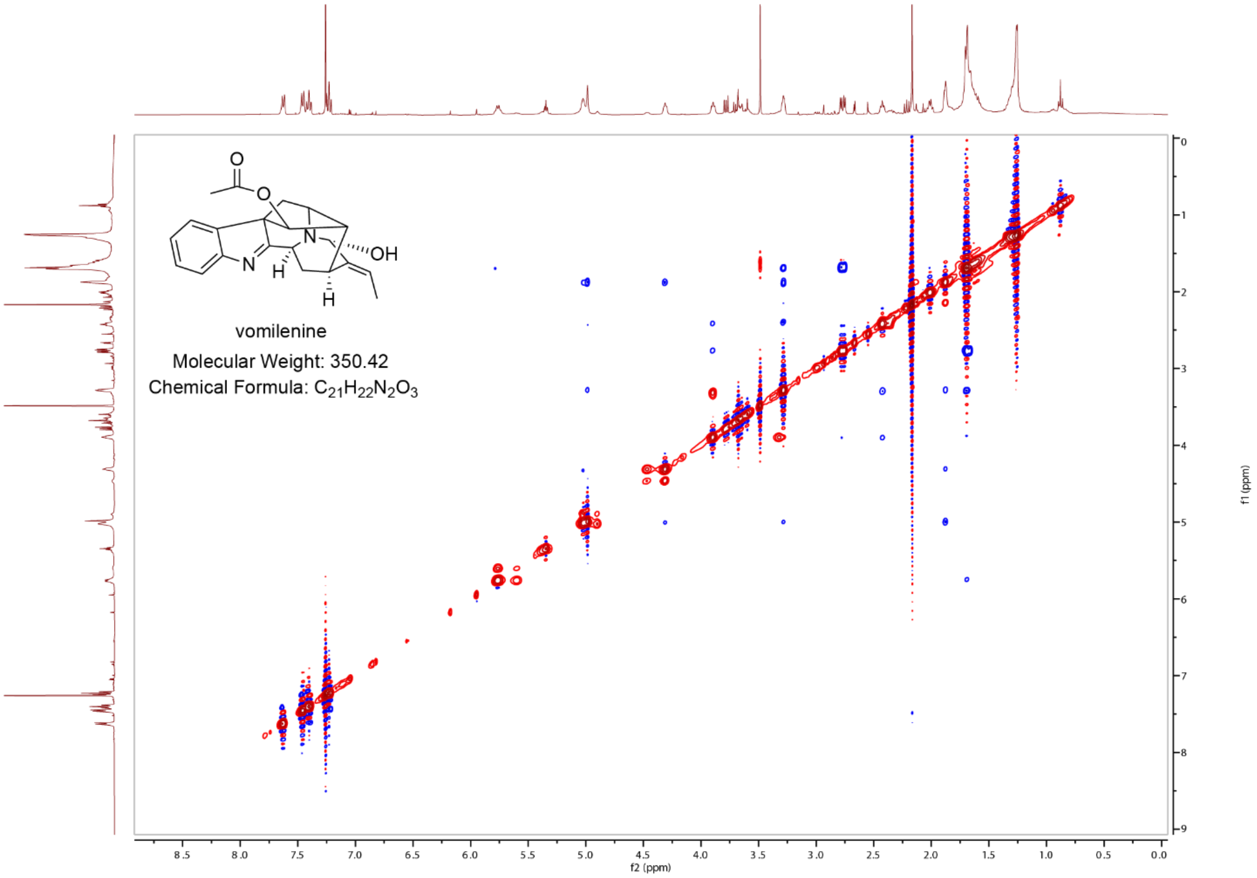

Supplementary Figure 7. **NOSEY spectra of vomilenine in $CDCl_3$.**

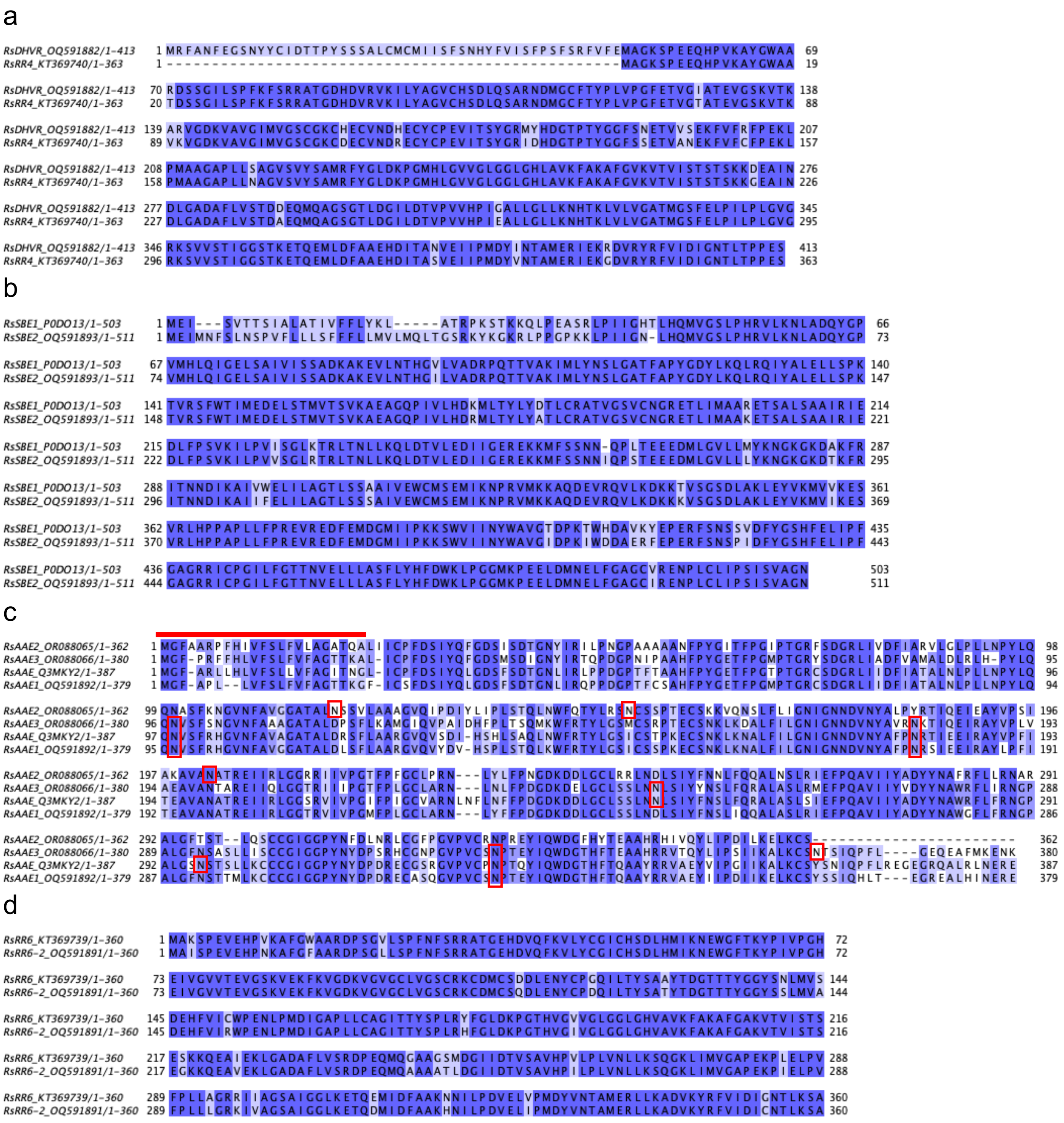

Supplementary Figure 8. Protein alignments showing the amino acid differences of enzymes discovered in this study and in previous literatures. (**a**) DHVR, (**b**) SBE, (**c**) AAE, and (**d**) RR6. The red line in (**c**) indicates the *N*-terminal secretory signal peptides of RsAAE and RsAAE1-3 as predicted by TargetP 2.0 (https://services.healthtech.dtu.dk/services/TargetP-2.0/). The red rectangles indicate putative *N*-glycosylation Asn residues as predicted by NetNGlyc-1.0 (https://services.healthtech.dtu.dk/services/NetNGlyc-1.0/). The Genbank numbers are: *RsDHVR* (this study, OQ591882); *RsRR4* (KT369740); *RsSBE1*(CYP71AY4, P0DO13); *RsSBE2* (this study, OQ591893); *RsAAE* (Q3MKY2); *RsAAE1* (this study, OQ591892); *RsAAE2* (this study, OR088065); *RsAAE3* (this study, OR088066); *RsRR6* (KT369739); *RsRR6-2* (this study, OQ591891).

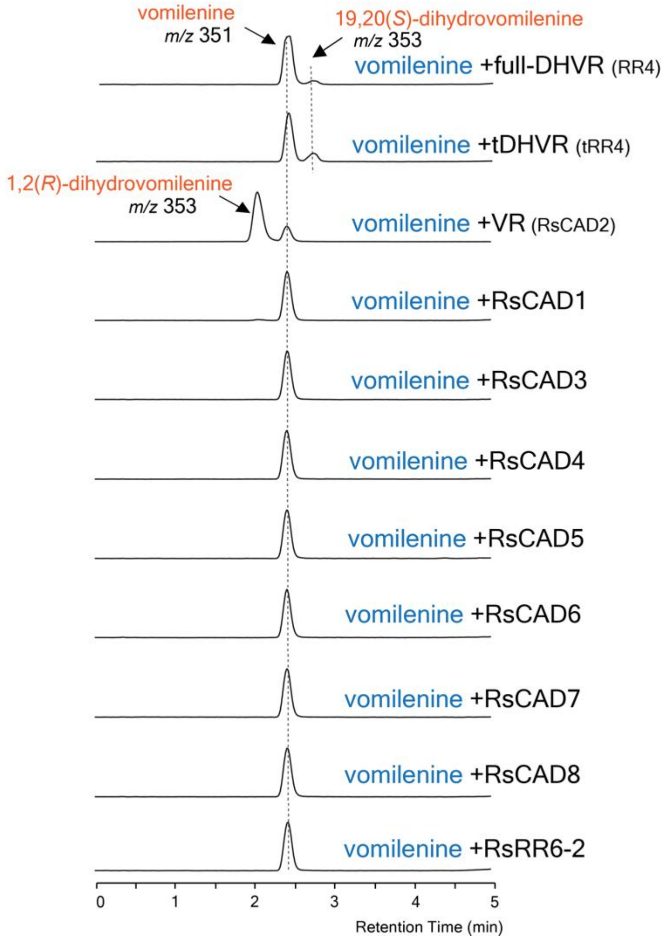

Supplementary Figure 9. *In vivo* screening identified RsCAD2 as the vomilenine 1,2-reductase. Vomilenine was fed to *Escherichia coli* cultures expressing candidate cinnamyl alcohol dehydrogenase (CAD)-like reductases (1-8), full-DHVR (95% identical to the reported VR2 at amino acid level), N-terminal truncated DHVR (tVR2), and RsRR6-2 (93% identical to the reported RsRR6 at amino acid level). Both full and truncated DHVR converted small amounts of the substrate vomilenine to 19,20(*S*)-dihydrovomilenine, while only VR (RsCAD2) showed vomilenine 1,2(*R*)-reductase activity. The LC-MS/MS chromatograms show combined ESI-MRM ion transitions of $[M+H]^+$ *m/z* 351→246, 353→82, 353→204, 355→80, 313→130, and 327→144 for detecting vomilenine, 1,2-dihydrovomilenine, 19,20-dihydrovomilenine, 17-*O*-acetylnorajmaline, norajmaline, and ajmaline, respectively. The ESI-MS/MS fragmentation patterns used to generate the MRM parameters can be found in Supplementary Figure 2.

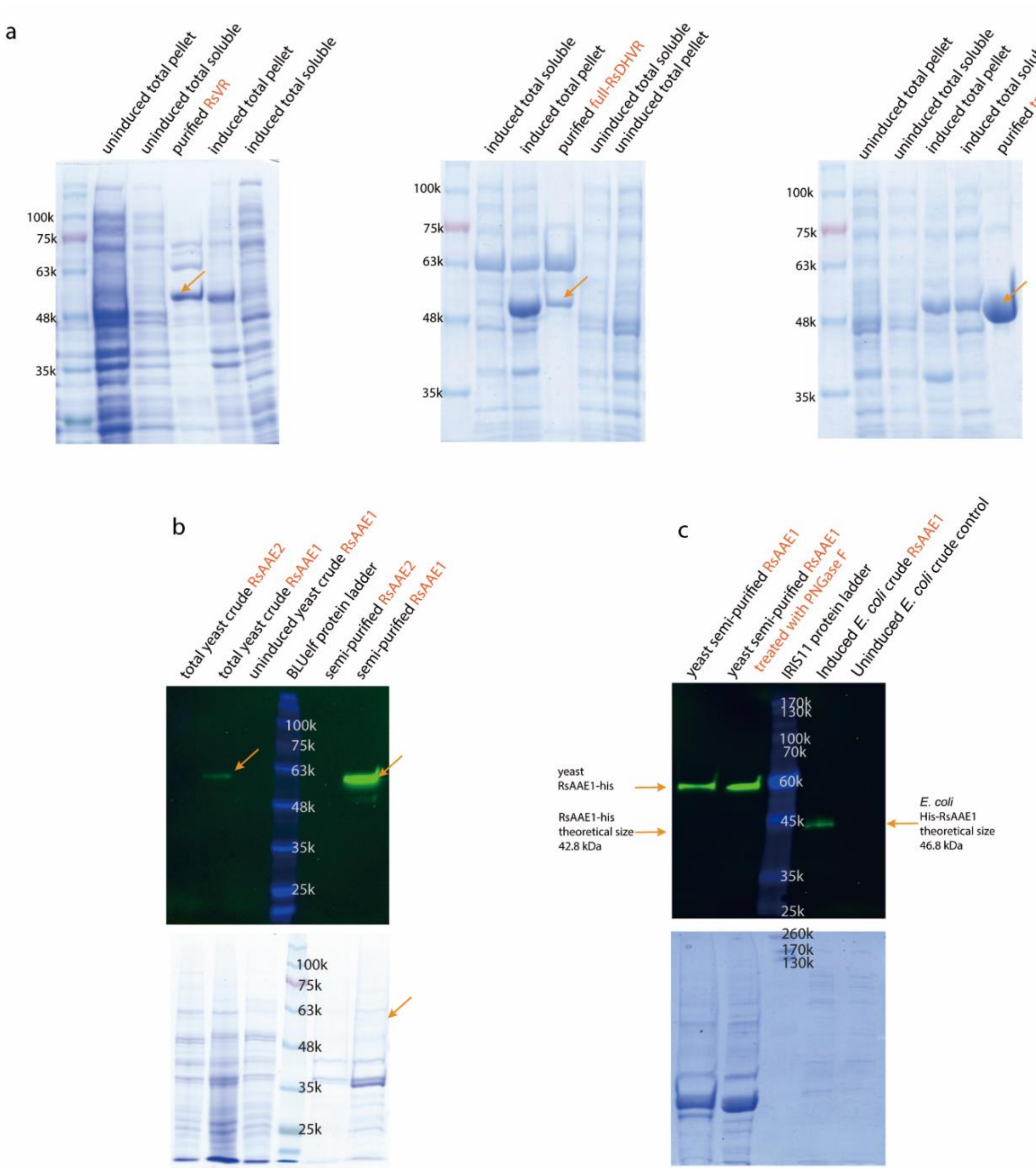

Supplementary Figure 10. SDS-PAGE and Western blot experiments for purified, his-tagged recombinant proteins for RsVR, full-length RsDHVR, N-terminal truncated RsDHVR, and RsAAE1 and 2. The recombinant proteins are indicated by orange arrows. (**a**) SDS-PAGE images of His-tagged recombinant protein purifications of RsVR (left panel), full-RsDHVR (middle panel), and N-terminal truncated RsDHVR (right panel) using standard Ni-NTA affinity chromatography from *E. coli*; (**b**) C-terminally His-tagged RsAAE1 was detected by anti-his antibody by Western blot in both crude yeast and Ni-NTA semi-purified RsAAE1 protein preparation (upper panel). The lower panel shows a replicate SDS-PAGE gel for protein loading. (**c**) *N*-terminally His-tagged RsAAE1 expressed in *E. coli* aligned with its theoretical sizes in Western blot, while the yeast recombinant AAE1 showed larger apparent molecular mass in the same blot (upper panel). Treating yeast recombinant RsAAE1 with PNGase F that removes the *N*-glycans did not significantly change AAE mobility in the blot. The lower panel is the same SDS-PAGE gel stained after the proteins were transferred to the membrane for blotting. The SDS-PAGE gels and blots were generated from a single experiment without replicates. Source data are provided as a Source Data file.

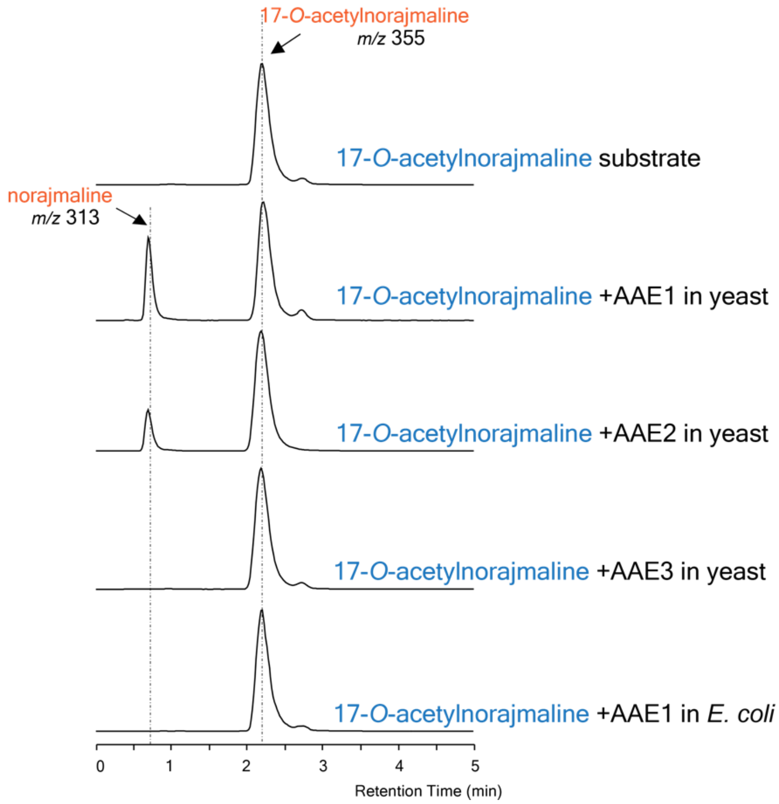

Supplementary Figure 11. Yeast expressing RsAAE1 and RsAAE2 converted 17-*O*-acetylnorajmaline to norajmaline *in vivo*. In comparison, RsAAE3 expressed in yeast or RsAAE1 expressed in *E. coli* did not show 17-*O*-acetylnorajmaline esterase activity. The expression of RsAAE1 in *E. coli* and yeast were confirmed by Western blot in Supplementary Figure 10b and 10c.

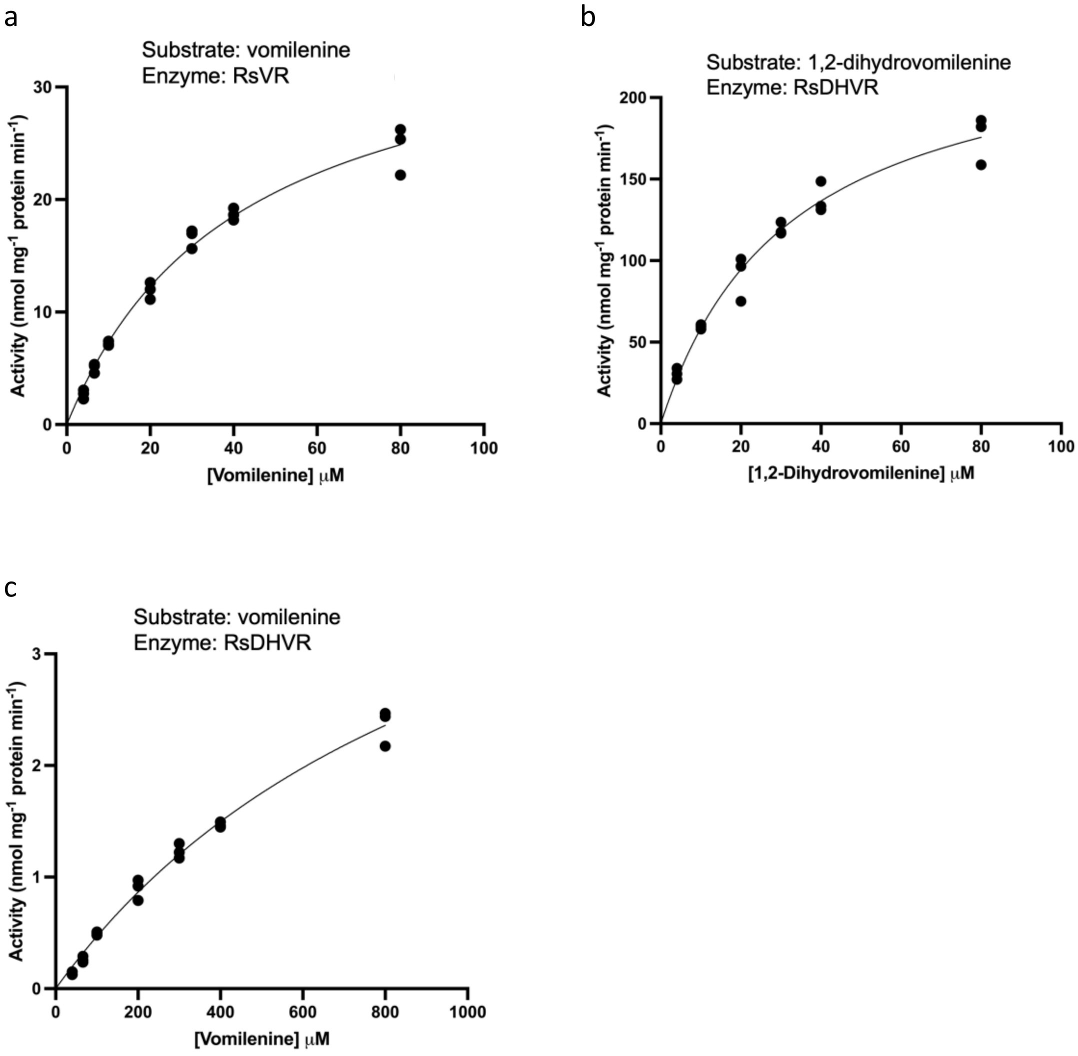

Supplementary Figure 12. Michaelis-Menten kinetics of RsVR and RsDHVR with vomilenine and/or 1,2(*R*)-dihydrovomilenine substrates. (**a**) RsVR kinetics with vomilenine substrate; (**b**) RsDHVR kinetics with 1,2(*R*)-dihydrovomilenine; (**c**) RsDHVR kinetics with vomilenine. RsVR does not take 19,20-dihydrovomilenine as substrate. Data was generated from triplicates (•) at each substrate concentration and graphed using Graphpad Prism 9.5.0. Source data are provided as a Source Data file.

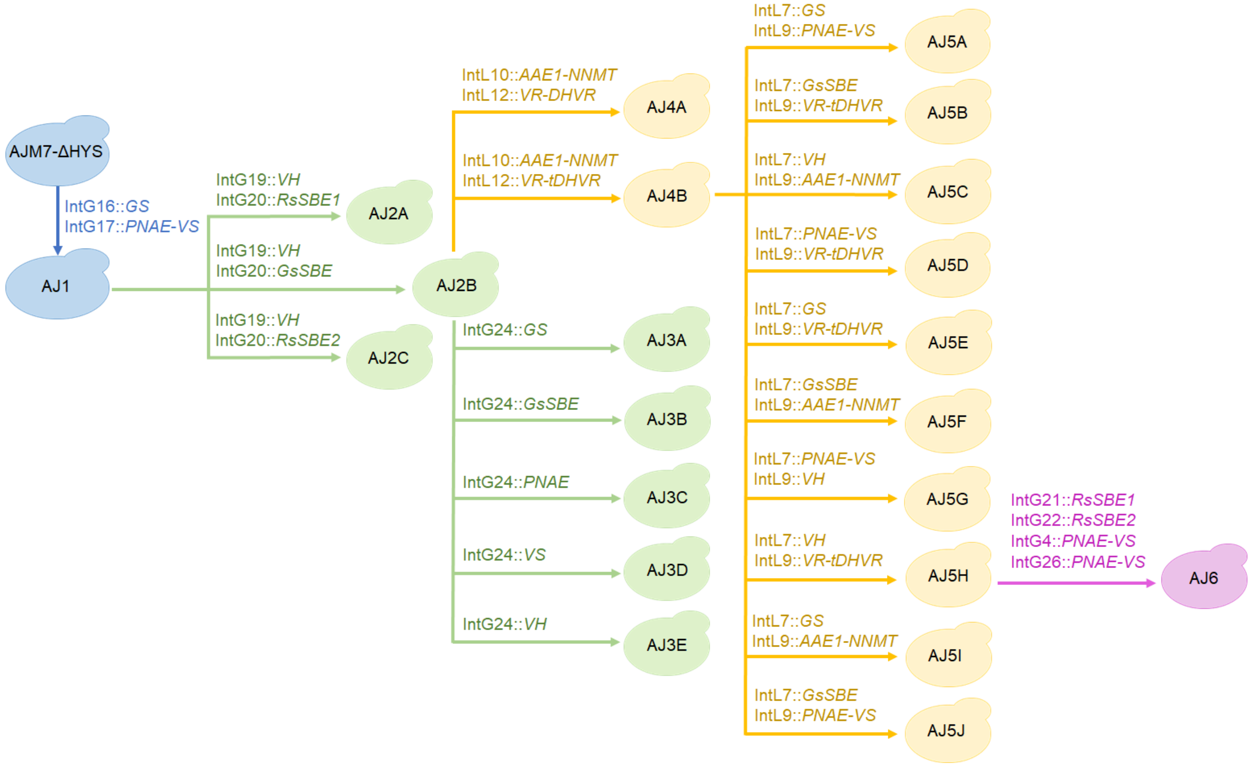

Supplementary Figure 13. ***S. cerevisiae*** **strains construction procedures.** Each round of genome editing was detailed including the gene cassettes integrated and the corresponding integration sites. The blue yeasts represent the chassis strains; green yeasts represent the strains capable of *de novo* biosynthesizing vomilenine; orange yeasts represent the strains with complete ajmaline biosynthetic pathway; the purple yeast represents the final strain achieving *de novo* biosynthesis of ajmaline.

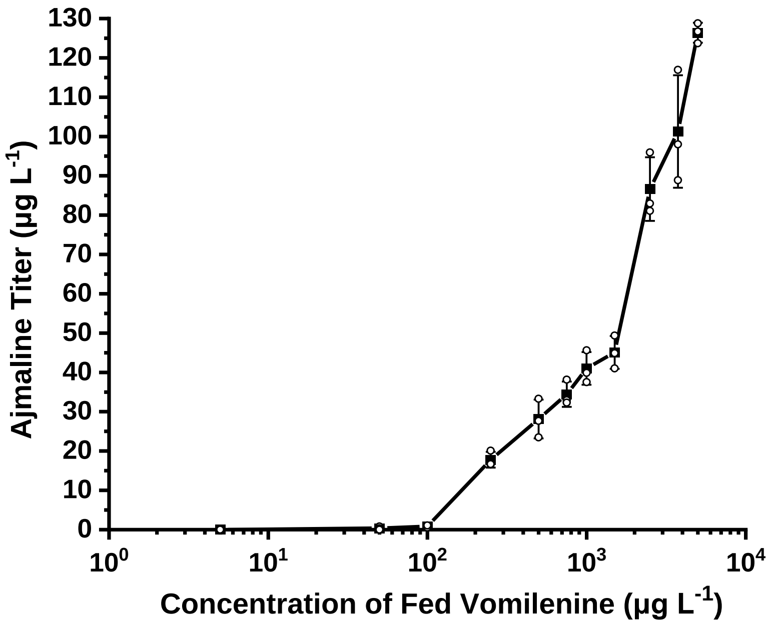

Supplementary Figure 14. The titer of ajmaline in strain AJ5H fed with different concentrations of vomilenine (ranging from 5 to 5,000 µg L^{-1}). When the concentration of additionally fed vomilenine was less than 100 µg L^{-1}, no significant accumulation of ajmaline was observed. Nevertheless, ajmaline was accumulated to significant levels when more than 250 µg L^{-1} of vomilenine was fed into the fermentation broth. The results verified that *de novo* biosynthesis of ajmaline was limited by the supply of vomilenine. The results represent the mean ± s.d. of biological triplicates (*n*=3) and were graphed using OriginPro 2021 9.8.0.200 software. Source data are provided as a Source Data file.

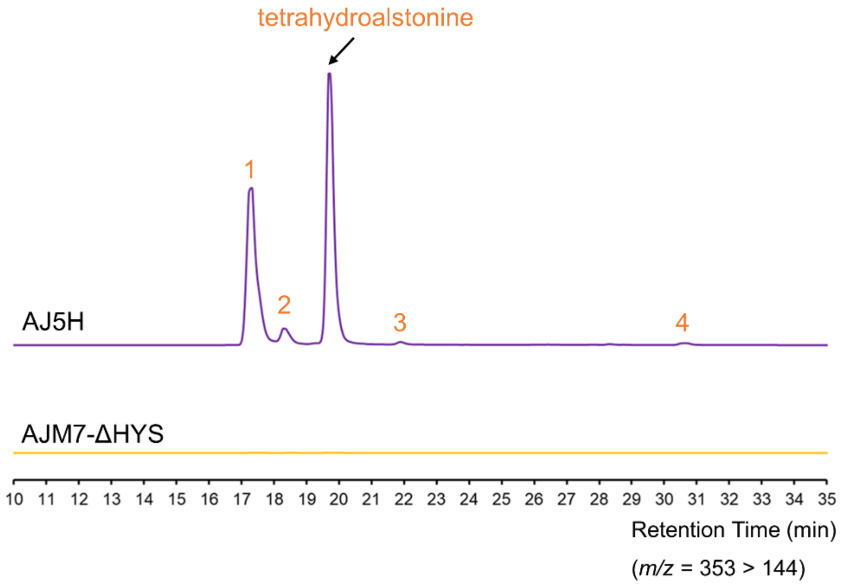

Supplementary Figure 15. LC-MS/MS analysis for fermentation sample of strains AJ5H and AJM7-ΔHYS. MRM spectra (*m/z*=353>144) of tetrahydroalstonine (by-product of GS) and four unknown by-products (number 1-4).

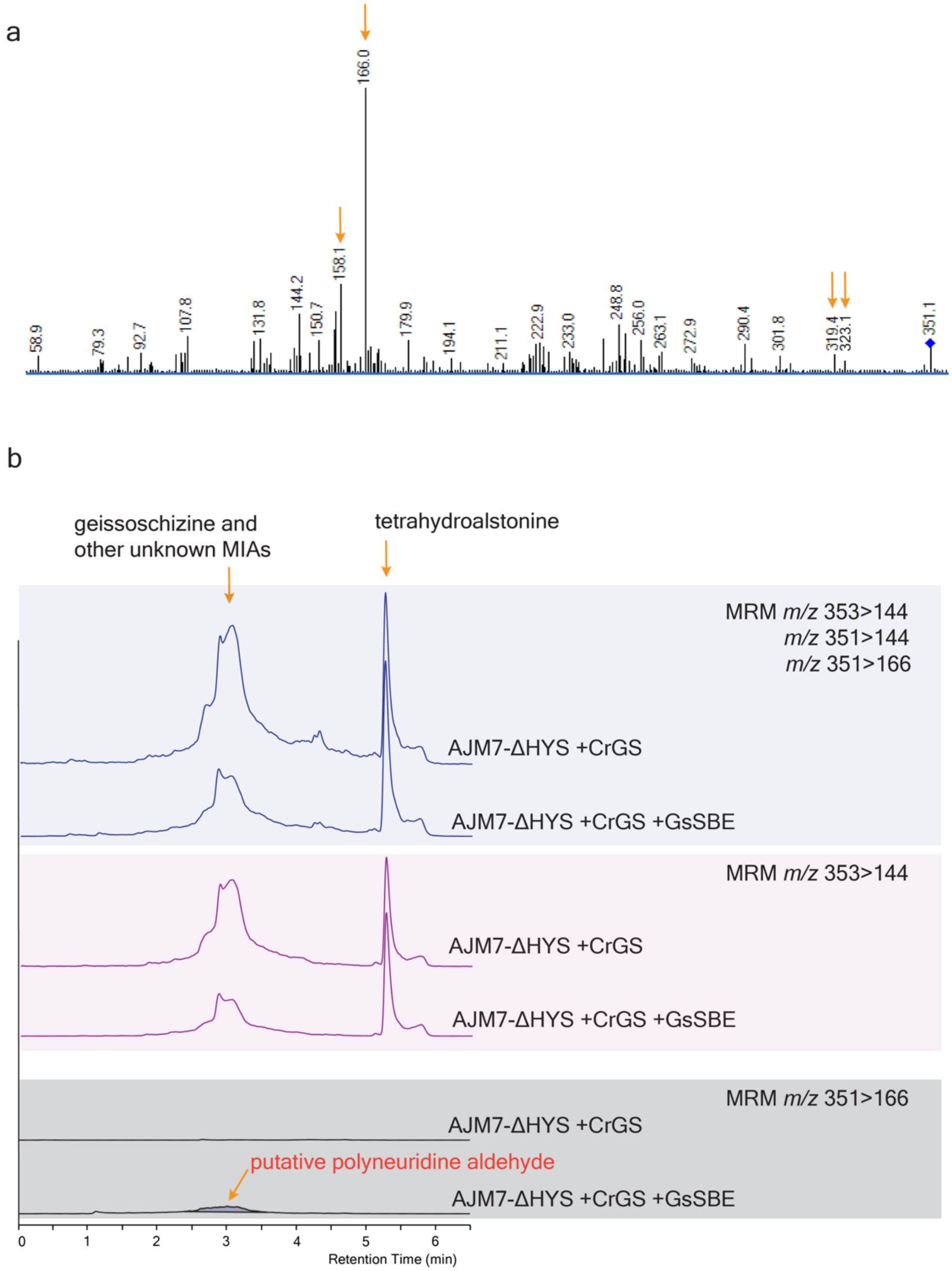

Supplementary Figure 16. Detection of low amounts of putative polyneuridine aldehyde intermediate in yeast culture expressing GS and SBE on multi-copy pESC vectors in synthetic complete medium. (**a**) The MS/MS product ion scans of putative polyneuridine aldehyde showed daughter ions *m/z* 323, 319, 166, and 158 that were consistent with reported daughter ions for polyneuridine aldehyde [2]. (**b**) The putative polyneuridine aldehyde accumulated in low amounts only when SBE was expressed and was not detected in GS only control yeast. MRM *m/z* 351>166 was used for detecting the putative polyneuridine aldehyde, whereas tetrahydroalstonine, geissoschizine, and other m/z 353 byproducts were detected with *m/z* 353>144 ion transitions. The chromatograms are under the same scale.

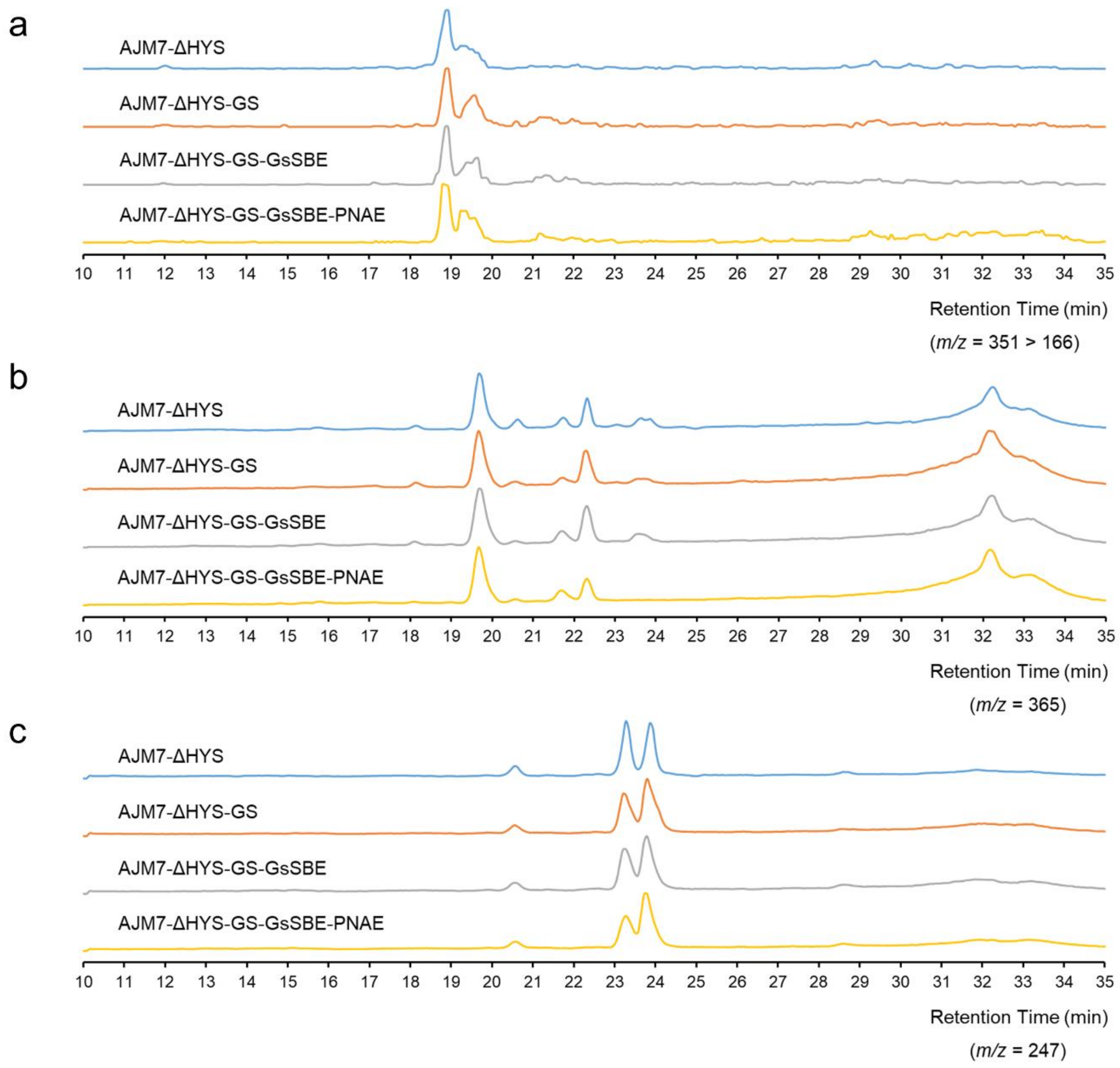

Supplementary Figure 17. LC-MS/MS analysis for fermentation sample (in YP medium) of yeast strains with ajmaline biosynthetic pathway genes integrated into the yeast genome. (**a**) MRM *m/z* 351>166 was used for detecting the putative polyneuridine aldehyde. (**b**) SIM *m/z* 365 was used for detecting the oxidative product of polyneuridine aldehyde (a hemiacetal). (**c**) SIM *m/z* 247 was used for detecting the degradation product of polyneuridine aldehyde (an aromatized MIA flavopepeirine). When a single copy of *GS*, *GS-GsSBE*, and *GS-GsSBE-PNAE* were integrated into the genome of AJM7-ΔHYS, respectively, LC-MS/MS profiles of these strains showed almost no difference, indicating no accumulation of putative polyneuridine aldehyde or its oxidation and degradation products.

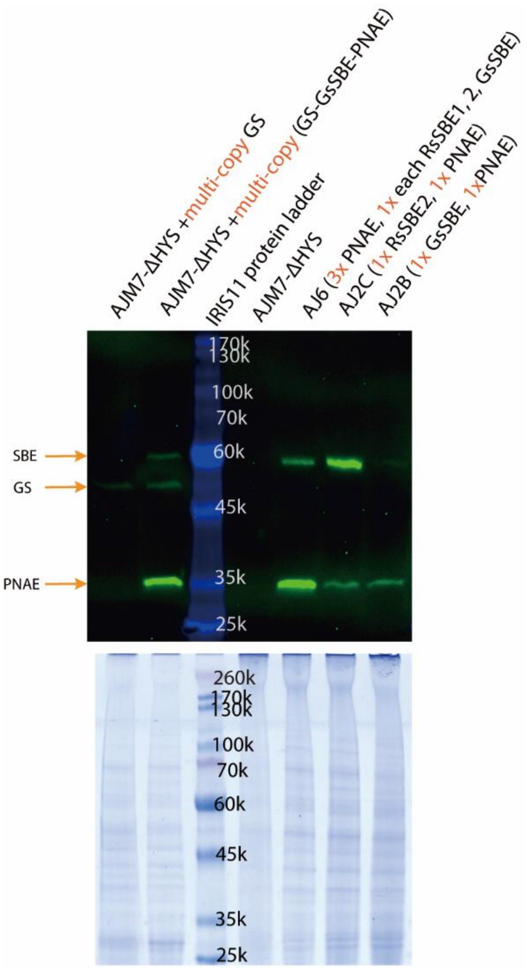

Supplementary Figure 18. Western blot analysis of the expression of C-terminal cMyc-tagged GS, SBE, and PNAE in different yeast strains. The details of each yeast strain can be found in Supplementary Figure 13. Both SBE and PNAE expression levels were enhanced in the final ajmaline-producing strain AJ6 (1 copy of each RsSBE1, RsSBE2, and GsSBE as well as 3 copies of PNAE) when compared with its parental strain AJ2B (1 copy of GsSBE and 1 copy of PNAE). While AJ2C had higher SBE expression level, both AJ2B and AJ2C accumulated comparable amounts of vomilenine (Figure 4c). Expressing C-terminal cMyc-tagged GS, GsSBE, and PNAE in the parental AJM7-ΔHYS strain on multi-copy pESC vectors showed higher expression levels than GsSBE and PNAE expressed as single genomic copy in strain AJ2B. GS integrated into the yeast genome did not contain the *C*-terminal cMyc-tag, thus strains AJ2B, AJ2C, and AJ6 did not show signal for GS. The lower panel shows a replicate SDS-PAGE gel for protein loading. The SDS-PAGE gels and blots were generated from a single experiment without replicates. Source data are provided as a Source Data file.

Supplementary Table 1. ^{1}H and ^{13}C chemical shifts of vomilenine in $CDCl_3$.

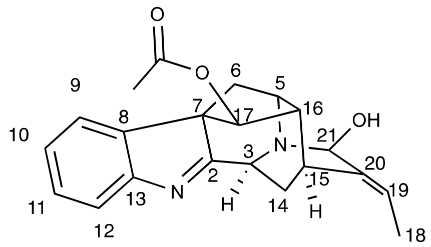

Chemical Formula: $C_{21}H_{22}N_2O_3$
Molecular Weight: 350.42

Carbon/ Proton #	^{1}H, this Study	^{1}H, Dang *et al.* [2]	^{13}C, this Study	^{13}C, Dang *et al.* [2]
**2**	-	-	182.5	181.7
**3**	4.32 (dd)	4.39 (br s)	54.5	n.d.
**5**	3.90 (dd)	3.93 (br s)	51.2	n.d.
**6**	2.78 (dd), 1.67 (m)	2.79 (dd), 1.71 (m)	36.8	36.2
**7**	-	-	65.3	65.0
**8**	-	-	136.2	136.1
**9**	7.46 (d)	7.47 (d)	123.9	123.8
**10**	7.23 (t)	7.24 (ddd)	128.9	125.7
**11**	7.41 (t)	7.41 (ddd)	125.8	128.9
**12**	7.62 (d)	7.64 (d)	121.2	121.2
**13**	-	-	156.3	156.5
**14**	1.88 (m)	1.90 (m)	26.6	26.2
**15**	3.28 (m)	3.33 (t)	28.6	28.0
**16**	2.43 (dd)	2.48 (t)	49.4	48.8
**17**	4.98 (s)	4.99 (s)	77.6	77.4
**18**	1.69 (s)	1.73 (dd)	13.3	13.2
**19**	5.76 (q)	5.8 (br q)	119.6	120.2
**20**	-	-	139.4	138.6
**21**	5.02 (br s)	5.12 (s)	82.6	83.1
**CO**	-	-	169.6	169.9
**CH_3CO**	2.17 (s)	2.18 (s)	21.2	21.1

Supplementary Table 2. Top 11 highly expressed cinnamyl alcohol dehydrogenase (CAD)-like reductases and other ajmaline biosynthetic genes in *Rauvolfia serpentina* root and leaf transcriptomes.

Query	Gene length	Name	Root TPM	Leaf TPM
TRINITY_DN464_c0_g1_i1	1702	*RsGS*	2600.9	483.3
TRINITY_DN9553_c0_g1_i28	1627	*RsCAD1*	1471.2	224.1
TRINITY_DN204_c0_g1_i53	1721	*RsVR (CAD2)*	730.7	0
TRINITY_DN9370_c1_g1_i1	1692	*RsCAD3*	594.3	84.3
TRINITY_DN4810_c0_g1_i8	2218	*RsDHVR (RR4)*	335.3	3.7
TRINITY_DN9063_c0_g1_i3	1358	*RsCAD4*	470.6	42.0
TRINITY_DN352_c0_g1_i1	1785	*RsRR6-2*	199.9	1.3
TRINITY_DN352_c0_g1_i9	1868	*RsCAD5*	109.1	11.4
TRINITY_DN6753_c0_g1_i4	1667	*RsCAD6*	116.5	75.6
TRINITY_DN204_c0_g1_i56	1637	*RsCAD7*	66.1	43.1
TRINITY_DN2461_c0_g1_i8	1495	*RsCAD8*	57.2	0.6
TRINITY_DN8466_c0_g2_i2	1495	*RsAAE2*	192.2	0.0
TRINITY_DN1015_c0_g1_i46	1623	*RsAAE3*	82.7	0.0
TRINITY_DN1015_c0_g1_i48	1678	*RsAAE1*	47.3	45.9
TRINITY_DN9500_c0_g1_i2	2109	*Rs8HGO*	472.6	85.4
TRINITY_DN4053_c0_g1_i1	2098	*RsG10H*	600.6	10.0
TRINITY_DN28264_c0_g1_i1	1528	*RsIS*	410.4	1.8
TRINITY_DN5042_c0_g1_i1	1874	*Rs7DLS*	1324.4	113.9
TRINITY_DN6254_c0_g1_i1	2003	*Rs7DLGT*	300.8	58.2
TRINITY_DN5048_c0_g1_i9	1947	*Rs7DLH*	274.8	94.1
TRINITY_DN288_c4_g1_i1	1687	*RsLAMT*	727.3	47.5
TRINITY_DN25782_c0_g1_i1	1844	*RsTDC*	444.9	36.9
TRINITY_DN4565_c1_g1_i3	1943	*RsSLS*	1024.0	117.0
TRINITY_DN9977_c0_g2_i1	1436	*RsSTR*	674.8	139.4
TRINITY_DN2262_c1_g1_i30	2017	*RsSGD*	186.9	21.9
TRINITY_DN351_c1_g1_i7	3200	*RsSBE2*	809.6	33.6
TRINITY_DN3800_c0_g1_i2	1225	*RsPNAE*	345.5	103.7
TRINITY_DN1152_c0_g1_i4	1476	*RsVS*	969.7	263.9
TRINITY_DN1379_c1_g1_i2	2356	*RsVH*	578.1	61.6
TRINITY_DN7737_c0_g3_i3	1284	*RsNNMT*	587.3	6.5

Supplementary Table 3. List of plasmids harboring ajmaline biosynthetic pathway genes constructed in this study.

Plasmid	Description
pESC-URA-*GS*	2μ; *URA3*; *AmpR*; *GAL1p-GS-CYC1t*
pESC-URA-*GsSBE*	2μ; *URA3*; *AmpR*; *GAL1p-GsSBE-CYC1t*
pESC-LEU2d-*RsSBE2-CrCPR*	2μ; *LEU2d*; *AmpR*; *GAL1p-RsSBE2-CYC1t*; *GAL10p-CrCPR-ADH1t*
pESC-URA-*RsSBE1*	2μ; *URA3*; *AmpR*; *GAL1p-RsSBE1-CYC1t*
pESC-HIS-*RsPNAE-RsVS*	2μ; *HIS3*; *AmpR*; *GAL1p-RsPNAE-CYC1t*; *GAL10p-RsVS-ADH1t*
pESC-LEU2d-*RsVH-CrCPR*	2μ; *LEU2d*; *AmpR*; *GAL1p-RsVH-CYC1t*; *GAL10p-CrCPR-ADH1t*
pESC-URA-*RsVH*	2μ; *URA3*; *AmpR*; *GAL10p-RsVH-ADH1t*
pESC-TRP-*RsAAE1*	2μ; *TRP1*; *AmpR*; *GAL10p-RsAAE1-ADH1t*
pESC-TRP-*RsNNMT-RsAAE1*	2μ; *TRP1*; *AmpR*; *GAL1p-RsNNMT-CYC1t*; *GAL10p-RsAAE1-ADH1t*
pESC-URA-*RsVR*	2μ; *URA3*; *AmpR*; *GAL10p-RsVR-ADH1t*
pESC-URA-*DHVR-RsVR*	2μ; *URA3*; *AmpR*; *GAL1p-DHVR-CYC1t*; *GAL10p-RsVR-ADH1t*
pESC-URA-*tDHVR-RsVR*	2μ; *URA3*; *AmpR*; *GAL1p-tDHVR-CYC1t*; *GAL10p-RsVR-ADH1t*
pESC-URA-*RsAAE1*	2μ; *URA3*; *AmpR*; *GAL10p-RsAAE1-ADH1t*
pESC-URA-*RsAAE2*	2μ; *URA3*; *AmpR*; *GAL10p-RsAAE2-ADH1t*
pESC-URA-*RsAAE3*	2μ; *URA3*; *AmpR*; *GAL10p-RsAAE3-ADH1t*

Supplementary Table 4. List of genome loci for the integration of ajmaline biosynthesis pathway gene cassettes.

Integration site	Spacer sequences (5'-3')	Chromosomal locus
IntL7	AATCCGAACAACAGAGCATA	ChrXVI: 776,883-776,902
IntL9	GCGCCACAGTTTCAAGGGTC	ChrXIV: 280,250-280,269
IntL10	CGCCATTCAAGAGCAGCAAC	ChrX: 236,843-236,862
IntL12	TTGTCACAGTGTCACATCAG	ChrXII: 839,660-839,679
IntG4	CCTGGCGCTATGATGATGAG	ChrVII: 478,898-478,917
IntG16	TATATAATGAATACACATGG	ChrVIII: 121,948-121,967
IntG17	GAAATTATATAAAACACATG	ChrVIII: 147,100-147,119
IntG19	AGATCTTGCGAAATACTGGG	ChrXV: 87,244-87,263
IntG20	TAATCAGTCTAACACCCCGG	ChrXV: 79,783-79,802
IntG21	TCAAGGGGTTGCATATAGGG	ChrXV: 73,653-73,672
IntG22	TCACACGAATGAGAATTGGG	ChrXV: 371,041-371,060
IntG24	AATCGGGGCAGACTATTCCG	ChrXV: 550,742-550,761
IntG26	GAGAAAATAAAAAAAATATG	ChrXV: 724,860-724,879

Supplementary references

1. Eurlings, M. C. M. *et al.* Forensic identification of indian snakeroot (*Rauvolfia serpentina* Benth. ex Kurz) using DNA barcoding. *J. Forensic Sci.* **58**, 822–830 (2013).

2. Dang, T.-T. T. *et al.* Sarpagan bridge enzyme has substrate-controlled cyclization and aromatization modes. *Nat Chem Biol* **14**, 760–763 (2018).
