# Supplementary material for: De novo biosynthesis of antiarrhythmic alkaloid ajmaline
Source: Nat Commun. 2024 Jan 11;15:457. doi: 10.1038/s41467-024-44797-z (PMC10784492; doi:10.1038/s41467-024-44797-z)
Supplement: Supplementary file 3 — Description of Additional Supplementary Files [file 41467_2024_44797_MOESM3_ESM.pdf]

Description of Additional Supplementary Files

Title: Supplementary Data 1

Description: Primers used in this study.

Title: Supplementary Data 2

Description: The protein sequences and Genbank accession numbers for Figure 3.
