# Supplementary material for: De novo biosynthesis of antiarrhythmic alkaloid ajmaline
Source: Nat Commun. 2024 Jan 11;15:457. doi: 10.1038/s41467-024-44797-z (PMC10784492; doi:10.1038/s41467-024-44797-z)
Supplement: Supplementary file 6 — Reporting Summary [file 41467_2024_44797_MOESM6_ESM.pdf]

nature portfolio

Corresponding author(s): Lian Jiazhang, Yang Qu

Last updated by author(s): Oct 24, 2023

Reporting Summary

Nature Portfolio wishes to improve the reproducibility of the work that we publish. This form provides structure for consistency and transparency in reporting. For further information on Nature Portfolio policies, see our Editorial Policies and the Editorial Policy Checklist.

Statistics

For all statistical analyses, confirm that the following items are present in the figure legend, table legend, main text, or Methods section.

n/a	Confirmed	
☐	☒	The exact sample size (*n*) for each experimental group/condition, given as a discrete number and unit of measurement
☐	☒	A statement on whether measurements were taken from distinct samples or whether the same sample was measured repeatedly
☒	☐	The statistical test(s) used AND whether they are one- or two-sided<br>*Only common tests should be described solely by name; describe more complex techniques in the Methods section.*
☒	☐	A description of all covariates tested
☒	☐	A description of any assumptions or corrections, such as tests of normality and adjustment for multiple comparisons
☐	☒	A full description of the statistical parameters including central tendency (e.g. means) or other basic estimates (e.g. regression coefficient) AND variation (e.g. standard deviation) or associated estimates of uncertainty (e.g. confidence intervals)
☒	☐	For null hypothesis testing, the test statistic (e.g. *F*, *t*, *r*) with confidence intervals, effect sizes, degrees of freedom and *P* value noted<br>*Give P values as exact values whenever suitable.*
☒	☐	For Bayesian analysis, information on the choice of priors and Markov chain Monte Carlo settings
☒	☐	For hierarchical and complex designs, identification of the appropriate level for tests and full reporting of outcomes
☒	☐	Estimates of effect sizes (e.g. Cohen's *d*, Pearson's *r*), indicating how they were calculated

Our web collection on statistics for biologists contains articles on many of the points above.

Software and code

Policy information about availability of computer code

Data collection	LC-MS/MS data was collected with Agilent Acquisition 10.0 software and Shimadzu LabSolutions 5.91 Software. NMR data was collected with Agilent VNMR OVJ2.1 software
Data analysis	LC-MS/MS data was analyzed with Agilent Qualitative Analysis 10.0 and Shimadzu LabSolutions 5.91 software. NMR data was analyzed with MestReNova v14.2.1 software. Protein sequence alignments (Muscle alignment) and phylogeny studies were analyzed using Mega version 11.0.10. The kinetics data was analyzed using Graphpad Prism 9 version 9.5.0. The RNA-seq data was analyzed using CLC genomic workbench 20.0.4. The bar graphs were generated with OriginPro 2021 9.8.0.200 software.

For manuscripts utilizing custom algorithms or software that are central to the research but not yet described in published literature, software must be made available to editors and reviewers. We strongly encourage code deposition in a community repository (e.g. GitHub). See the Nature Portfolio guidelines for submitting code & software for further information.

Data

Policy information about availability of data

All manuscripts must include a data availability statement. This statement should provide the following information, where applicable:

- Accession codes, unique identifiers, or web links for publicly available datasets
- A description of any restrictions on data availability
- For clinical datasets or third party data, please ensure that the statement adheres to our policy

The primers are listed in Supplementary Data 1. The protein sequences for Figure 3 are available in Supplementary Data 2. The RNA-seq for reads are available at NCBI-SRA ([https://www.ncbi.nlm.nih.gov/sra/?term=SRR26495086] and [https://www.ncbi.nlm.nih.gov/sra/?term=SRR26495085]). Source data are provided with this paper.

Research involving human participants, their data, or biological material

Policy information about studies with human participants or human data. See also policy information about sex, gender (identity/presentation), and sexual orientation and race, ethnicity and racism.

Reporting on sex and gender	N/A
Reporting on race, ethnicity, or other socially relevant groupings	N/A
Population characteristics	N/A
Recruitment	N/A
Ethics oversight	N/A

Note that full information on the approval of the study protocol must also be provided in the manuscript.

Field-specific reporting

Please select the one below that is the best fit for your research. If you are not sure, read the appropriate sections before making your selection.

☒ Life sciences ☐ Behavioural & social sciences ☐ Ecological, evolutionary & environmental sciences

For a reference copy of the document with all sections, see nature.com/documents/nr-reporting-summary-flat.pdf

Life sciences study design

All studies must disclose on these points even when the disclosure is negative.

Sample size	Following similar biochemistry and metabolic engineering studies in literature, we used three replicates that are sufficient in providing mean values and representing data variation.
Data exclusions	No data were excluded
Replication	Three biological replicates were used. All attempts at replication were successful.
Randomization	Yeast colonies were randomly picked from the agar plates. No other randomization was used in this study, since they are not applicable for enzyme characterization or metabolic engineering experiments.
Blinding	No blinding was used in this study. Blinding is not applicable to enzyme kinetics studies or metabolic engineering experiments.

Reporting for specific materials, systems and methods

We require information from authors about some types of materials, experimental systems and methods used in many studies. Here, indicate whether each material, system or method listed is relevant to your study. If you are not sure if a list item applies to your research, read the appropriate section before selecting a response.

Materials & experimental systems

n/a	Involved in the study	
☐	☒	Antibodies
☒	☐	Eukaryotic cell lines
☒	☐	Palaeontology and archaeology
☒	☐	Animals and other organisms
☒	☐	Clinical data
☒	☐	Dual use research of concern
☐	☒	Plants

Methods

n/a	Involved in the study	
☒	☐	ChIP-seq
☒	☐	Flow cytometry
☒	☐	MRI-based neuroimaging

Antibodies

Antibodies used

His-Tag Antibody (H-3), mouse monoclonal, cat#sc-8036, Santa Cruz Biotechnology Inc., Dallas, Texas, USA; cMyc antibody (9E10), mouse monoclonal, cat# sc-40, Santa Cruz Biotechnology, Inc., Dallas, Texas, USA; Donkey anti-Mouse IgG (H+L) Cross-Adsorbed Secondary Antibody, HRP (cat#SA1-100), ThermoFisher Scientific

Validation

Both the two primary antibodies are from commercial sources. The validation is provided by the manufactures:

1. His-Tag Antibody (H-3) is a mouse monoclonal IgG1 κ His-Tag antibody (also designated 6X His-Tag antibody or His-Probe antibody) that detects the His-Tag protein, cited in 508 publications, provided at 200 µg/ml.
Reference: Camel single-domain antibodies as modular building units in bispecific and bivalent antibody constructs. | Els Conrath, K., et al. 2001. J Biol Chem. 276: 7346-50. PMID: 11053416

2. Myc Antibody (9E10) is a mouse monoclonal IgG1 κ Myc antibody, cited in 9,595 publications, provided at 200 µg/ml. It is raised against an epitope corresponding to amino acids 408-439 within the C-terminal domain of c-Myc of human origin.
Reference: Transcriptional activation by the human c-Myc oncoprotein in yeast requires interaction with Max. | Amati, B., et al. 1992. Nature. 359: 423-6. PMID: 1406955

Dual use research of concern

Policy information about dual use research of concern

Hazards

Could the accidental, deliberate or reckless misuse of agents or technologies generated in the work, or the application of information presented in the manuscript, pose a threat to:

No	Yes	
☒	☐	Public health
☒	☐	National security
☒	☐	Crops and/or livestock
☒	☐	Ecosystems
☒	☐	Any other significant area

Experiments of concern

Does the work involve any of these experiments of concern:

No	Yes	
☒	☐	Demonstrate how to render a vaccine ineffective
☒	☐	Confer resistance to therapeutically useful antibiotics or antiviral agents
☒	☐	Enhance the virulence of a pathogen or render a nonpathogen virulent
☒	☐	Increase transmissibility of a pathogen
☒	☐	Alter the host range of a pathogen
☒	☐	Enable evasion of diagnostic/detection modalities
☒	☐	Enable the weaponization of a biological agent or toxin
☒	☐	Any other potentially harmful combination of experiments and agents

Plants

Seed stocks	Rauvolfia serpentina plant in this study was a clonal propagation of the plant used in the PhytoMetaSyn project. The species has been identified by plant morphology and the intron sequence of rps16 gene.
Novel plant genotypes	N/A
Authentication	N/A
